# The bidirectional association between sleep problems and autism spectrum disorder: a population-based cohort study

**DOI:** 10.1186/s13229-018-0194-8

**Published:** 2018-01-30

**Authors:** Maria E. Verhoeff, Laura M. E. Blanken, Desana Kocevska, Viara R. Mileva-Seitz, Vincent W. V. Jaddoe, Tonya White, Frank Verhulst, Maartje P. C. M. Luijk, Henning Tiemeier

**Affiliations:** 1 The Generation R Study Group, Erasmus Medical Center, Rotterdam, the Netherlands; 2 Department of Child and Adolescent Psychiatry/Psychology, Erasmus University Medical Center–Sophia Children’s Hospital, 2060, Rotterdam, 3000 CB the Netherlands; 3 Department of Pediatrics, Erasmus University Medical Center–Sophia Children’s Hospital, Rotterdam, the Netherlands; 4 Department of Epidemiology, Erasmus University Medical Center, Rotterdam, the Netherlands; 5 Department of Radiology, Erasmus University Medical Center, Rotterdam, the Netherlands; 6 Department of Psychology, Education and Child Studies, Erasmus University Rotterdam, Rotterdam, the Netherlands; 7 Department of Psychiatry, Erasmus University Medical Center, Rotterdam, the Netherlands

**Keywords:** Autism, Sleep problems, Bidirectional, Birth cohort, General population

## Abstract

**Background:**

Sleep difficulties are prevalent in children with autism spectrum disorder (ASD). The temporal nature of the association between sleep problems and ASD is unclear because longitudinal studies are lacking. Our aim is to clarify whether sleep problems precede and worsen autistic traits and ASD or occur as a consequence of the disorder.

**Methods:**

Repeated sleep measures were available at 1.5, 3, 6, and 9 years of age in 5151 children participating in the Generation R Study, a large prospective birth cohort in the Netherlands. Autistic traits were determined with the Pervasive Developmental Problems score (PDP) of the Child Behavior Checklist (CBCL) at 1.5 and 3 years and the Social Responsiveness Scale (SRS) at 6 years. This cohort included 81 children diagnosed with ASD.

**Results:**

Sleep problems in early childhood were prospectively associated with a higher SRS score, but not when correcting for baseline PDP score. By contrast, a higher SRS score and an ASD diagnosis were associated with more sleep problems at later ages, even when adjusting for baseline sleep problems. Likewise, a trajectory of increasing sleep problems was associated with ASD.

**Conclusions:**

Sleep problems and ASD are not bidirectionally associated. Sleep problems do not precede and worsen autistic behavior but rather co-occur with autistic traits in early childhood. Over time, children with ASD have an increase in sleep problems, whereas typically developing children have a decrease in sleep problems. Our findings suggest that sleep problems are part of the construct ASD.

**Electronic supplementary material:**

The online version of this article (10.1186/s13229-018-0194-8) contains supplementary material, which is available to authorized users.

## Background

Autism spectrum disorder (ASD) affects 0.5 to 1% of children [1–3] and has an early onset, typically before age 2 [4–6]. ASD is often characterised by severe deficiencies in social interaction and communication, accompanied by repetive behaviour. Children with ASD frequently suffer from comorbid psychopathologies [1, 7–9]. Among those, sleep problems, defined as difficulties falling asleep or nightmares, are common [10] occurring in 40–80% of cases across all ages [11–17] in comparison to 25–50% in normally developing children [16, 18, 19]. The broad range of prevalence estimates is explained by multiple factors, such as different measures for sleep problems, age of the child, IQ of the autistic children studied, and the heterogeneity of ASD. What is more, there is no clear definition of clinically relevant sleep problems in pediatric populations, resulting in various forms of research questions on sleep problems in ASD (for a review on prevalence of sleep problems in ASD see Richdale & Schreck, 2009) [16]. As mentioned, the type of sleep problems differs; younger children with ASD exhibit more bedtime resistance, bedtime anxiety, awakenings during the night, and parasomnias (defined as abnormal behavior during sleep, such as sleep walking, sleep talking, and nightmares), whereas older children mainly exhibit insomnia symptoms (defined as the difficulty falling asleep or staying asleep) [16, 20].

The association between sleep problems and ASD can be of two forms. First sleep problems may precede and worsen the behavioral outcome of ASD [20–22]. Second, sleep problems occur as a consequence of the underlying disorder**.** Sleep problems are common in early childhood with prevalence estimates of up to 50% [18]; prevalence decline in typically developing children but not in children with ASD [23]. Risk factors or correlates of early childhood sleep problems are, for example, maternal psychopathology, parenting practices, child temperament, difficulties setting bedtime, and feeding patterns [19, 24, 25]. The influence of these factors diminishes when the child’s sleep patterns become more stable [24, 25]. Studies have indicated that children with ASD have more sleep problems than typically developing children [16]. A British cohort study showed that children with and without ASD have similar sleep durations in infancy, but from 30 months onwards, their sleep is characterized by a shorter duration than typically developing children [26]. Another study showed that children with autistic traits developed more sleep problems in pre-adolescence [27]. However, most previous studies of sleep problems in children with ASD are cross-sectional and the few longitudinal studies have a lack of baseline measures and diagnosis of ASD [21, 27–29]. Thus, it is difficult to properly asses the course of sleep problems in children with ASD [28, 30, 31]. To unravel the complex temporal nature of the association between sleep problems and ASD, it is essential to have prospective research that measures autistic traits and associated sleep problems repeatedly throughout childhood.

In this study, we explored the association between the onset of sleep problems and autistic traits and ASD in the general population. An ASD diagnosis must be confirmed by a licensed clinician, while autistic traits are autistic symptoms that do not meet the diagnostic criteria for ASD assessed by questionnaires. Repeated assessments of autistic traits and sleep problems were obtained at several developmental stages. It is important to clarify whether sleep problems precede and worsen autistic traits and ASD or occur after (other) symptoms of ASD become manifest. This enables us to gain more insight in the course over time of sleep problems in children with ASD.

First, we expected that the onset of sleep problems precedes and worsens the early manifestations of autistic traits. Second, we hypothesized that sleep problems in children with autistic traits or with ASD emerge early in life and increase over time.

## Methods

### Design and study population

This study was embedded in Generation R, a prospective population-based cohort from fetal life onward [32]. All pregnant women (expected delivery date April 2002–January 2006) living in Rotterdam, the Netherlands, were invited to participate by their midwife or obstetrician during routine visits. The participation rate was estimated at 61%. We obtained written informed consent from all participants and their parents. The Medical Ethical Committee of the Erasmus Medical Center Rotterdam approved the study.

**Fig. 1:**
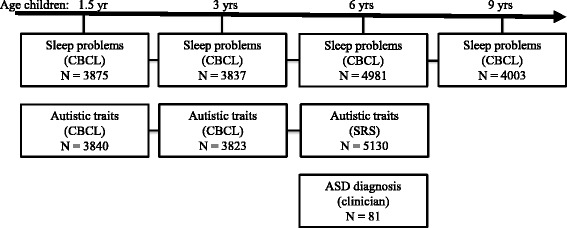
Population and measurements overview

Data on sleep problems based on at least one assessment from age 1.5 year onward were available for 7464 children. Children without information on autistic traits or a diagnosis of ASD were excluded (*n* = 2313), yielding a sample size of 5151 children for the present study (follow-up rate 69.0%). In the analyses, the study population varies slightly due to missing data in different assessments rounds (see Fig. 1 for study overview).

### Sleep problems

Children’s sleep problems were quantified using the Sleep Problem Scale, which is a pre-defined Problem Scale of the Child Behavior Checklist (CBCL), a reliable and valid measure for behavioral problems [33, 34]. The CBCL is widely used internationally and has been found to be generalizable across 23 societies [35]. The CBCL was completed by the primary caregiver, in the majority of cases, the mother, who rated various sleep problems of the child in the previous 2 months on a three-point Likert scale (0 = not true, 1 = somewhat true, 2 = very true).

The Sleep Problem Scale compromises seven questions about sleep problems including items on dyssomnia and parasomnia. For ages 1.5, 3, and 6, the Sleep Problem Scale was based on CBCL for ages 1.5–5. This scale has previously been used as a measure of sleep problems [36–39]. At older ages, the CBCL differs slightly to adapt to developmental changes throughout childhood. Because there is not an established subscale for measuring sleep problems at age 9, we a priori selected 5 items from the CBCL 6–18 questionnaires based on the appropriateness to measure sleep problems and ran an exploratory factor analysis in order to construct a scale for sleep problems at 9 years, resulting in a two-factor solution dyssomnia and parasomnia with an internal consistency of *α* = 0.55. The low internal consistency is most likely due to the two-factor solution combined in 1 sleep problem scale. As our interest is assessing general sleep problems, we decided to keep a combined scale rather than separate dyssomnia and parasomnia scales. This sleep problem scale has previously been used in the same format as a measure of sleep problems [40].

### Autistic traits

Autistic traits were measured twice with different instruments**.** First, at ages of 1.5 and 3 years, we used the Pervasive Development Problem (PDP) scale of the CBCL 1.5–5 (see details about CBCL above) to assess autistic like trait/pervasive developmental problems as an indicator of autistic traits. We calculated the sum score of the PDP scale. Second, at the age of 6 years (range 5–8 years), the Social Responsiveness Scale (SRS) was administered to obtain a measure of autistic traits. The SRS provides a valid quantitative measure of subclinical and clinical autistic traits [41]. We utilized the 18-item short-form of the scale, containing three subscales: social cognition, social communication, and social mannerism. The subscales show correlations ranging from 0.93 to 0.99 with the full scale in three different large studies. The authors of the scale recommend cutoffs for screening in population-based settings (consistent with weighted scores of 1.078 for boys and 1.000 for girls) [41].

### ASD diagnosis

All diagnoses in our records were made by a licensed clinical psychologists or psychiatrists, these were only retrieved if diagnoses were formally coded according to the Diagnostic and Statistical Manual of Mental Disorders (DSM) IV/5 or the International Classification of Primary Care (ICPC). ASD diagnoses were retrieved from general practitioners. In the Dutch health care system, all specialists are obliged to inform the general practitioner as the primary health care provider, who holds the central medical records. The following steps were performed to select children with high sensitivity for potentially retrieving a clinician-made ASD diagnosis. We selected children with one of the three following indicators for a further diagnostic work-up of ASD. First, all children, who scored in the top 15th percentile on the CBCL for ages 1.5–5 total score or those in the top 2nd percentile on the PDP subscale, were screened with the Social Communication Questionnaire (SCQ), a 40-item parent-reported screening instrument for ASD [42]. We retrieved medical records of all children, who scored positive on the SCQ. Second, we also retrieved medical records of all children, who scored above the cutoff 1.078 for boys and 1.000 for girls on the SRS-short form. Third, we retrieved medical records of all children whose mother at any moment, in a questionnaire or a research center interview, up to age 8 years had reported that the child had undergone a diagnostic procedure for possible ASD.

Only children for whom a diagnosis of ASD could be confirmed by specialist medical records before age 9 were classified as ASD in the analyses. The specialist diagnoses of ASD were generally based on clinical consensus by a multidisciplinary team. The standard diagnostic work-up typically involves an extensive developmental case history obtained from parents, as well as school information, and repeated observations of the child. The mean age of diagnosis of ASD in our sample was 6 years, for convenience hereafter referred to as ASD diagnosis at 6 years (range 2–9 years). See Additional file 1: Table S1 for correlations between all measures of autistic trait and ASD.

### Covariates

Based on literature, covariates were included if they were an antecedent of ASD and/or were associated with sleep problems, but not consequences or intermediates such as somatic complains. The following variables were considered possible confounders in the association between sleep problems and autistic traits and ASD [24, 43]. Sex and gestational age of the children were obtained from the medical records completed by community midwives and obstetricians. In accordance with Central Bureau for Statistics, child ethnicity was based on country of birth of the parents, which was assessed by questionnaire and coded as, Dutch, Other-Western, and non-Western. Information on maternal characteristics was obtained by questionnaire during pregnancy. Maternal education was defined by the highest attained educational level and classified into three categories (low, middle, and high education) in line with the definition of Central Bureau for Statistics [44]. Finally, maternal psychopathology, such as anxiety, depressive symptoms, hostility, and psychoticism, was assessed prenatally using the Brief Symptom Inventory (BSI) [45].

### Statistical analysis

First, we tested sleep problems as predictors of continuously measured autistic traits with linear regression and as predictors of the dichotomous ASD diagnosis with logistic regression. Second, we analyzed the prospective association of autistic traits and ASD with sleep problems using linear regression.

Subsequently, to compare defined groups of children based on their patterns of sleep disturbances over time, the association of the latent class trajectories with autistic traits and ASD were analyzed using linear regression and logistic regression. Trajectories of sleep disturbance up to 6 years of age were defined using Latent Class Trajectory Models [46], as previously applied to this data in Generation R [47]. This is a person-centered modeling approach that estimates growth curves over time across unobserved subpopulations by assigning a most likely latent trajectory class to each individual. The three-class model as previously defined in this cohort fit our sample best [47]. The largest group was the “decreasing to low sleep disturbances” class (*n* = 2423, 47.0%), which followed a normative developmental decline of sleep problems and was therefore defined as the reference. The “stable at medium sleep disturbances” class comprised 1318 (25.6%) children and the “increasing to high sleep disturbances” class comprised 622 (12.1%) children.

We constructed two models for all analyses. In the first model, the following confounders were included: child sex, gestational age, ethnicity, maternal psychopathology, and maternal educational level. To test the temporal direction of the association between ASD and sleep problems, we additionally adjusted all models for baseline PDP score or baseline Sleep Problem Score. For ease of comparison over the different instruments and time points, we used *z*-transformed versions of all independent and dependent variables, except for the ASD diagnosis in all analyses. To reduce bias associated with missing data, we used multiple imputations for missing values of the covariates. Ten imputed datasets were created and analyzed separately after which the results were pooled [48]. A sensitivity analysis was conducted to test the robustness of our findings. We repeated the analyses excluding children with a diagnosis of ASD, representing the most “severe” cases of ASD, using only autistic traits as exposure or determinant. The statistical analyses were performed using the SPSS version 22.0 for Windows (IBM Corp., Armonk, NY, USA) and MPlus version 7.11 (Muthén & Muthén, Los Angeles, CA, USA) using Monte Carlo integration techniques and maximum likelihood estimation with robust standard errors.

## Results

Characteristics of the children with and without ASD are presented in Table 1. Children with a diagnosis of ASD (*n* = 81) were more often boys (86.4%). Their mothers reported more psychopathological symptoms for themselves than mothers of children without ASD. There were no significant differences in the other characteristics, such as maternal age at birth.

**Table 1:** Characteristics of the study population

	*N*	No ASD diagnosis *N* = 5062	ASD diagnosis *N* = 81
Child characteristics			
Gender (% girls)	5143	50.1	13.6*
Gestational age at birth (weeks)	5102	39.80 (0.03)	39.36 (0.26)*
Ethnicity (%)			
Dutch	3386	69.7	75.6
Other-Western	461	9.5	7.7
Non-Western	1007	20.8	16.7
Sleep problem score			
At 1.5 years	3875	1.51 (0.03)	1.75 (0.26)
At 3 years	3837	1.50 (0.03)	1.77 (0.24)
At 6 years	4981	1.02 (0.02)	1.85 (0.25)*
At 9 years	4003	0.82 (0.02)	1.92 (0.28)*
Trajectories of sleep problems (%)			
Increasing sleep problems	622	15.6	33.8*
Decreasing sleep problems	2432	54.8	43.7*
Stable medium sleep problems	1318	29.6	22.5
Autistic traits			
PDP score––1.5 years	3840	1.77 (0.03)	2.26 (0.23)*
PDP score––3 years	3823	2.03 (0.03)	4.81 (0.44)*
SRS score––6 years	5130	0.22 (0.00)	0.97 (0.08)*
Abdominal pain (%)	5064	7.5	5.7
Functional constipation (%)	4894	3.5	7.5
Maternal Characteristics			
Age at inclusion (years)	5143	31.4 (0.1)	31.0 (0.5)
Educational level (%)			
No education/primary school	275	5.7	2.6
High school/lower vocational training	1885	38.6	51.3*
Higher vocational or academic training	2699	55.7	46.2
Psychopathology score	5295	0.24 (0.00)	0.38 (0.07)*

Data represent means (SDs) unless specified otherwise

*Abbreviations*: *PDP* Pervasive Development Problem scale, *SRS* Social Responsiveness Scale

*p < 0.05

We first tested the cross-sectional association between sleep problems and autism. At all ages, they were significantly associated (e.g., sleep problems at age 1.5 years and autistic traits, *B* = 0.27, 95% CI 0.23 to 0.31, *p < 0.01*).

### The longitudinal association of sleep problems with autistic traits and ASD

Table 2 shows the longitudinal associations of sleep problems with autistic traits and ASD adjusted for covariates and baseline autistic traits. Children who presented sleep problems at 1.5 and 3 years were more likely to have autistic traits. However, after adjusting for baseline PDP score, no longitudinal association was observed between sleep problems and autistic traits.

**Table 2:** The longitudinal association of sleep problems with autistic traits and autism spectrum disorder

		Autistic traits* at 3 years			Autistic traits** at 6 years			ASD at 6 years		
Sleep problems		*B*	95% CI	*p*	*B*‡	95% CI	*p*	OR	95% CI	*p*
1.5 years										
	Model 1	0.12	0.08–0.15	< 0.01	0.08	0.05–0.11	< 0.01	1.11	0. 87–1.42	0.41
	Model 2	0.03	− 0.01–0.06	0.13	0.03	− 0.02–0.06	0.07	1.05	0. 81–1.35	0.73
3 years										
	Model 1	0.20	0. 17–0.23	< 0.01	0.07	0.04–0.10	< 0.05	1.11	0.86–1.43	0.43
	Model 2	–	–	–	0.01	− 0.03–0.04	0.70	0.95	0.72–1.24	0.70

Model 1 was adjusted for gender, ethnicity, gestational age, maternal education, and maternal psychopathology. Model 2 was additionally adjusted for prevalent autistic traits

*Abbreviations*: *ASD* autism spectrum disorder, *SRS* Social Responsiveness Scale

^‡^Because of the narrow distribution of the SRS, Bs are given in hundredth SRS points

*Measured with PDP-scale CBCL

**Measured with SRS score

### The longitudinal association of autistic traits and ASD with sleep problems

Table 3 shows the longitudinal associations of autistic traits at ages 1.5, 3, and 6 years, and also that of ASD at age 6 years with sleep problems at age 9 years, after adjustment for covariates and baseline sleep problems. We found a significant association between autistic traits at age 1.5 and 3 years were related to more sleep problems at 6 years, both unadjusted and adjusted for baseline sleep problems. Furthermore, children with autistic traits and children with ASD at 6 years had more sleep problems at 9 years.

**Table 3:** The longitudinal association of autistic traits and autism spectrum disorder with sleep problems

		Sleep problems at 6 years			Sleep problems at 9 years		
Autism measure		*B*	95% CI	*p*	*B*	95% CI	*p*
Autistic traits* 1.5 years							
	Model 1	0.13	0.09–0.16	< 0.01	0.10	0.06–0.14	< 0.01
	Model 2	0.07	0.03–0.10	< 0.01	0.08	0.03–0.12	< 0.01
Autistic traits* 3 years							
	Model 1	0.13	0.10–0.16	< 0.01	0.06	0.04–0.08	< 0.01
	Model 2	0.05	0.02–0.08	< 0.01	0.04	0.03–0.06	< 0.01
Autistic traits** 6 years							
	Model 1	0.13	0.10–0.16	< 0.01	0.14	0.10–0.18	< 0.01
	Model 2	–	–	–	0.11	0.07–0.14	< 0.01
ASD 6 years							
	Model 1	0.46	0.24–0.68	< 0.01	0.84	0.58–1.10	< 0.01
	Model 2	–	–	–	0.74	0.49–0.99	< 0.01

Model 1 was adjusted for gender, ethnicity, gestational age, maternal education, and maternal psychopathology. Model 2 was additionally adjusted for prevalent sleep problems

*Abbreviations*: *ASD* autism spectrum disorder, *PDP* Pervasive Development Problem scale, *SRS* Social Responsiveness Scale

*Measured with PDP-scale CBCL

**Measured with SRS score

### Sleep problem trajectories

Children with a trajectory of increasing sleep problems and children with stable and moderate sleep problems had higher levels of autistic traits than those with decreasing sleep problems (Additional file 1: Table S2). We found that an increasing course of sleep problems was consistently associated with ASD at age 6 years (Additional file 1: Table S2).

### Sensitivity analyses

All analyses were adjusted for gender, ethnicity, gestational age, maternal education, and maternal psychopathology, and, if possible, baseline measures of respectively sleep problems or prevalent autistic traits. There was no significant interaction between gender and sleep problems on the risk of autism or between gender and ASD in the analysis of sleep (data not shown).

Sensitivity analyses indicated our findings were robust. The results of all regression analyses remained unchanged after the children with ASD were excluded (data not shown).

## Discussion

In this large population-based cohort, we found that sleep problems in toddlerhood were associated with autistic traits in mid-childhood, but this association disappeared when adjusting for early autistic traits. In contrast, autistic traits and a diagnosis of ASD in childhood were associated with sleep problems at later ages. Consistently, children with increasing sleep problems across development were more likely to have autistic traits and ASD. Our findings suggest that sleep problems are part of the construct of ASD, however do not predict severity of autistic traits over time. We showed that there is no bidirectional relation between sleep problems and ASD.

Our finding that sleep problems are associated with more autistic traits is in line with previous studies [21, 28, 31]. However, these previous studies lacked the repeated measurements of sleep problems and autistic traits across ages [21, 31]. When we adjusted for baseline autistic traits in the current study, the association between sleep problems at younger ages and later autistic traits disappeared. We found no evidence for sleep problems preceding autistic traits at baseline. This implies that sleep problems do not predict autistic traits and ASD over and above symptoms such as diminished social and communicative abilities, which are measured by the PDP scale. Moreover, sleep problems do not worsen ASD.

Autistic traits and ASD were associated with more sleep problems in accordance with previous studies [28, 49, 50]. This association remained even after adjusting for baseline sleep problems. Sleep problems in young children are relatively common and can be considered part of normative development in the general population [26, 51]. Yet, as supported by our trajectory analyses, the severity and frequency of sleep problems decreases in typically developing children [47], whereas sleep problems worsen over time in children with ASD. This strongly suggests that the pathology underlying ASD on the behavioral sequelae determines the development of sleep problems.

The course of sleep problems over time in these children is poorly understood. Previous studies have been unable to determine the temporal association [31]. By using trajectories of sleep problems and relating the trajectories to autistic traits and ASD, we show that sleep problems tend to decrease and disappear in the general population but increase in children with ASD. These trajectories are a further indication that the longitudinal course of sleep problems is a symptom and consequence of ASD, rather than worsen ASD symptomatology [9]. Thus, sleep problems are prevalent in children with ASD and should be considered part of the disorder.

Children with ASD suffer from more sleep problems than children without ASD, but the pathophysiology of sleep problems in children with ASD has not yet been fully understood. Some studies point to underlying deficits in endogenous melatonin secretion [52], others to alterations in hypothalamic-pituitary adrenal-axis function and cortisol secretion [53, 54], alterations in neurodevelopmental pathways [55], and some to polygenetic variations in circadian rhythm and clock genes related to ASD pathology [56]. As ASD is highly heritable [57–61], it would be worthwhile to study whether there are shared underlying genetic factors between sleep problems and autistic traits. Another mechanism could be that social problems associated with ASD may worsen the day-night rhythm in these children [14] and play a crucial role in the development of sleep problems. Socialization of day-night rhythm, such as bedtime routines, nighttime rituals, and family regularity [62–64], are important in young children as they can act as social zeitgebers and thereby contribute to the development of a healthy sleep pattern and the prevention of the occurrence of sleep problems [14, 56, 65]. Children with ASD have difficulty to adequately respond to the social zeitgebers and therefore struggle to develop a healthy sleep pattern [56]. More research is needed to unravel the socialization of day-night rhythm in children with ASD and the linkage with the development of sleep problems. Future studies should emphasize bedtime routines and family regularity when investigating children with ASD and sleep problems.

Our findings indicate that sleep problems do not contribute to an exacerbation of autistic traits but rather that sleep problems manifest as part of the broad ASD symptomatology. This is important information for parents who worry sleep problems may precipitate or perpetuate autistic symptoms. Based on our findings, we underline the importance of addressing sleep problems in children with ASD, possibly in the context of ASD treatments. Whereas sleep problems do not have a direct effect on autistic traits, sleep problems are known to negatively affect daytime functioning, such as attention processes and executive functioning [66–68]. Executive dysfunction co-occurs with the core symptoms of ASD [69–71], but the pathways remain unclear. We speculate that sleep problems can contribute to the development of executive dysfunction in children with ASD, but this needs further research. Future research should also investigate the effect of treatment of sleep problems and its concurrent effects on neurocognitive outcomes in children with ASD.

### Strengths and limitations

The current study has relevant strengths. First, the large sample size and longitudinal design enabled us to study sleep problems and autistic traits at multiple ages across a broad time span and to control for baseline characteristics and confounders. Our longitudinal design also enabled us to account for reverse causality. Second, we were able to complement parent-reported autistic symptoms with a diagnosis of ASD.

The current study, however, also had some limitations. First, we used a mother-reported questionnaire to assess sleep problems. It would have been ideal to use actigraphic or polysomnographic measures for studying sleep problems. However, mothers are known to be reliable reporters of children’s sleep at younger ages [72]. Second, our earliest assessment of autistic traits was performed at 1.5 years. Although measuring autistic traits in the general population in younger children is not very reliable [73], mostly retrospective studies documented that many children who develop ASD will have had symptoms prior to age 1.5 years. As such, our earliest measurement may not represent the pathophysiological onset of the disorder. However, our study characterizes the nature of the longitudinal association between sleep and autistic traits in early childhood. Third, children with ASD were slightly more likely to be lost to follow-up than typically developing children. Nevertheless, our sensitivity analyses yielded similar results after excluding all ASD cases and were therefore likely not affected by the lost to follow-up. Fourth, we used different age-appropriate measures of autistic traits that had a low to moderate correlation at different ages. The effect of adjustment for baseline autistic traits may be influenced by using different measures. Nevertheless, when we adjusted for baseline autistic traits, the association between sleep problems and autistic traits disappeared. If anything, if adjustment with the same measure had been possible, this would have further attenuated any observed association.

## Conclusions

To conclude, we showed that sleep problems do not precede and worsen autistic behavior but rather co-occur with autistic traits in early childhood. Furthermore, sleep problems increase over time in children with ASD. Although sleep problems in young children are often considered part of normal development, our findings suggest that sleep problems persisting to later ages can be considered as symptoms of ASD.

## Additional file

Additional file 1: **Table S1.** Pearson’s correlations among measures of autistic traits and ASD diagnosis. **Table S2.** The longitudinal association of sleep problem trajectories with autistic traits and autism spectrum disorder. (DOCX 17 kb)

## Abbreviations

ASD: Autism spectrum disorder
BSI: Brief Symptom Inventory
CBCL: Child Behavior Checklist
DSM: Diagnostic and Statistical Manual of Mental Disorders
ICPC: International Classification of Primary Care
PDP: Pervasive Developmental Problems
SCQ: Social Communication Questionnaire
SRS: Social Responsiveness Scale
