# Supplementary material for: The bidirectional association between sleep problems and autism spectrum disorder: a population-based cohort study
Source: Mol Autism. 2018 Jan 30;9:8. doi: 10.1186/s13229-018-0194-8 (PMC5791216; doi:10.1186/s13229-018-0194-8)
Supplement: Additional file 1: — Table S1. Pearson’s correlations among measures of autistic traits and ASD diagnosis. Table S2. The longitudinal association of sleep problem trajectories with autistic traits and autism spectrum disorder. (DOCX 17 kb) [file 13229_2018_194_MOESM1_ESM.docx]

**Table S1.** Pearson’s correlations among measures of Autistic Traits and ASD diagnosis.

| Autism measure | Autistic traits*  1.5 years | Autistic traits*  3 years | Autistic traits**  6 years | ASD 6 years |
| --- | --- | --- | --- | --- |
| Autistic traits*  1.5 years |  |  |  |  |
|  | 1 | 0.47 | 0.32 | 0.05 |
| *p* |  | <0.01 | <0.01 | <0.05 |
| N | 5151 | 5151 | 5138 | 5143 |
| Autistic traits*  3 years |  |  |  |  |
|  |  | 1 | 0.40 | 0.15 |
| *p* |  |  | <0.01 | <0.01 |
| N |  | 5151 | 5138 | 5143 |
| Autistic traits**  6 years |  |  |  |  |
|  |  |  | 1 | 0.34 |
| *p* |  |  |  | <0.01 |
| N |  |  | 5138 | 5130 |
| ASD 6 years |  |  |  |  |
|  |  |  |  | 1 |
| *p* |  |  |  |  |
| N |  |  |  | 5143 |

Abbreviations: ASD: Autism Spectrum Disorder, PDP: Pervasive Development Problem scale SRS: Social Responsiveness Scale

* Measured with PDP-scale CBCL.

**Measured with SRS score.

**Table S2.**  The longitudinal association of sleep problem trajectories with autistic traits and Autism Spectrum Disorder

|  |  | | Autistic traits*  at 6 years | | | |  | | ASD  at 6 years | | |
| --- | --- | --- | --- | --- | --- | --- | --- | --- | --- | --- | --- |
| Sleep Trajectory | |  | | B‡ | 95% CI | *p* | |  | OR | 95% CI | *p* |
| Increasing course of sleep problems | |  | |  |  |  | |  |  |  |  |
|  | | Model 1 | | 0.29 | 0.20-0.37 | <0.01 | |  | 2.98 | 1.63 – 5.43 | <0.01 |
|  | | Model 2 | | 0.19 | 0.07-0.20 | <0.01 | |  | 2.39 | 1.29 – 4.43 | <0.01 |
| Stable course of medium sleep problems | |  | |  |  |  | |  |  |  |  |
|  | | Model 1 | | 0.14 | 0.10-0.27 | <0.01 | |  | 0.89 | 0.47 – 1.70 | 0.73 |
|  | | Model 2 | | 0.09 | 0.03-0.15 | <0.01 | |  | 0.78 | 0.40 – 1.51 | 0.46 |
| Decreasing course of sleep problems | |  | |  |  |  | |  |  |  |  |
|  | | Model 1 | | 0 | (ref.) |  | |  | 1.0 | (ref.) |  |
|  | | Model 2 | | 0 | (ref.) |  | |  | 1.0 | (ref.) |  |

Abbreviations: ASD: Autism Spectrum Disorder, SRS: Social Responsiveness Scale

‡ Because of the narrow distribution of the SRS, Bs are given in hundredth SRS points

*Autistic traits are measured with the SRS score

Model 1 was adjusted for gender, ethnicity, gestational age, maternal education, and maternal psychopathology. Model 2 was additionally adjusted for prevalent autistic traits.
